# Association of RANKL and EGFR gene expression with bone metastases in patients with metastatic non-small cell lung cancer

**DOI:** 10.3389/fonc.2023.1145001

**Published:** 2023-05-05

**Authors:** Anita J.W.M. Brouns, Lizza E.L. Hendriks, Iris J. Robbesom-van den Berge, Annemariek J.H.M. Driessen, Guido M.J.M. Roemen, Britt L.J. van Herpen, Zoë Dekkers, Bas Heitzer, Daphne J.G. Leunissen, Laura Moonen, Ragnar Lunde, Marcel Westenend, Marjolein van Driel, Ernst-Jan M. Speel, Anne-Marie C. Dingemans

**Affiliations:** ^1^ Department of Respiratory Medicine, Zuyderland, Geleen, Netherlands; ^2^ Department of Respiratory Medicine, Maastricht University Medical Center+, Maastricht, Netherlands; ^3^ Department of Pulmonary Diseases, GROW - School for Oncology and Reproduction, Maastricht University Medical Center, Maastricht, Netherlands; ^4^ Department of Internal Medicine, Erasmus MC, Rotterdam, Netherlands; ^5^ Department of Clinical Pharmacy and Toxicology, Maastricht University Medical Center+, Maastricht, Netherlands; ^6^ Department of Pathology, GROW-School for Oncology and Reproduction, Maastricht University Medical Center+, Maastricht, Netherlands; ^7^ Department of Respiratory Medicine, Laurentius Hospital, Roermond, Netherlands; ^8^ Department of Respiratory Medicine, Viecuri Medical Center, Venlo, Netherlands; ^9^ Department of Respiratory Medicine, Erasmus MC Cancer Institute, University Medical Center, Rotterdam, Netherlands

**Keywords:** bone metastases, receptor activator of nuclear factor κb ligand, epidermal growth factor expression, osteoprotegerin, lung adenocarcinoma, epidermal growth factor mutation

## Abstract

**Introduction:**

Bone metastases are frequent in patients with non-small cell lung cancer (NSCLC). The receptor activator of Nuclear Factor κB (RANK)/RANK ligand (RANKL)/osteoprotegerin (OPG) pathway is important in bone metastases development. Furthermore, epidermal growth factor receptor (EGFR) signaling promotes osteoclast formation and stimulation. The understanding of the biological mechanism of bone metastases development might have implications for treatment strategies. Therefore, we studied whether there is an association between EGFR, RANKL, RANK and OPG gene expression in the tumor and presence of bone metastases in patients with NSCLC.

**Methods:**

From an updated multicenter study, including patients with *EGFR* mutated (*EGFR*+), Kirsten rat sarcoma *(KRAS+)* and *EGFR/KRAS* wildtype metastatic NSCLC, all patients with available formalin-fixed paraffin-embedded (FFPE) tumor samples were selected. Ribonucleic Acid (RNA) was isolated from these samples and gene expressions of EGFR, RANKL, OPG and RANKL were determined *via* quantitative Polymerase Chain Reaction (qPCR). Data on demographics, histology and molecular subtyping, sample origin, presence of bone metastasis, SREs and bone progression were collected. Primary endpoint was relation between EGFR, RANK, RANKL, OPG gene expression, RANKL: OPG ratio and bone metastases.

**Results:**

In 73/335 (32% *EGFR+*, 49% *KRAS+*, 19% *EGFR/KRAS* wildtype) samples from unique patients, gene expression analysis could be performed. Of these 73 patients, 46 (63%) had bone metastases at diagnosis or developed bone metastases during the disease course. No association was found between EGFR expression and presence of bone metastases. Patients with bone metastases had a significantly higher RANKL expression and RANKL: OPG ratio compared to those without. An increased RANKL: OPG ratio resulted in a 1.65x increased risk to develop bone metastases, especially in the first 450 days after diagnosis of metastatic NSCLC.

**Conclusion:**

Increased RANKL gene expression and RANKL: OPG ratio, but not EGFR expression, was associated with presence of bone metastases. Additionally, an increased RANKL: OPG gene ratio was associated with a higher incidence of bone metastases development.

## 1 Introduction

The skeleton is a common site for tumor metastases of several malignancies. For example, 30-60% of patients with metastatic lung cancer develop bone metastases (1, 2). In patients with bone metastases, bone turnover is disturbed. Normal bone remodeling requires a perfect balance between osteoblasts, osteoclasts and numerous signaling pathways, growth factors and control mechanisms. An important role is reserved for the Receptor activator of Nuclear Factor κB (RANK)/RANK ligand (RANKL)/osteoprotegerin (OPG, the decoy receptor and antagonist of RANKL) pathway in bone development (3). By binding of RANKL to RANK, an ongoing cascade is set in motion, in which cancer cells stimulate osteoclasts, which in turn degrade the bone. During osteoclastogenic bone resorption different growth factors and cytokines are released from the bone, which stimulate the cancer cells to expansive growth (4).

Epidermal growth factor receptor (EGFR) signaling is involved in the proliferation of osteoclast precursors. Signaling *via* EGFR promotes osteoclast formation and stimulation by inhibition of OPG expression and by increasing monocyte chemoattractant protein 1 (MCP1; which induces osteoclast fusion and activity), macrophage colony-stimulating factor (MSCF) and RANKL expression (5, 6). An *in vitro* study showed that the addition of EGFR-tyrosine kinase inhibitors (EGFR-TKIs) completely blocked RANKL-dependent osteoclast formation and led to apoptosis in matured osteoclasts. These observations suggest an essential role for EGFR signaling in RANKL-mediated osteoclast differentiation and survival (7).

EGFR protein expression, determined by immunohistochemistry, in non-small cell lung cancer (NSCLC) is up-regulated in 40-80% of the tumors (8, 9). Conflicting results exist regarding the association of EGFR protein expression and *EGFR* mutations in NSCLC: some studies showed a higher EGFR protein expression in tumor samples (n=133-970) of patients with *EGFR* mutated (*EGFR+*) NSCLC (10, 11), while others (n=102-159) showed no association (12, 13). The up-regulated EGFR protein expression in the tumor (which possibly results in increased EGFR signaling) that was observed in some studies evaluating *EGFR+* NSCLC, could be an explanation for our previously reported higher incidence of bone metastases in *EGFR+* NSCLC compared with Kirsten rat sarcoma (*KRAS+*) and *EGFR/KRAS* wildtype NSCLC (14).

To the best of our knowledge, it has never been studied in a clinical setting whether there is an association between EGFR, RANKL, RANK and OPG gene expression in the tumor and presence of bone metastases in patients with NSCLC. In this study, we tried answering this question since understanding the biological mechanism of bone metastases development might have implications for adequate bone metastasis screening and (prophylactic) treatment decisions.

## 2 Materials and methods

Data from a study of patients with metastatic NSCLC were used (1). In this case-control study, for every patient with *EGFR*+ NSCLC (i.e., exon 19 deletion or exon 21 point mutation), the consecutive patients with a *KRAS*+ and *EGFR/KRAS* wildtype NSCLC were included as a case-control group. Wildtype was defined as *EGFR* and *KRAS* mutation negative NSCLC, as extensive molecular testing was not standard of care at that time. The established database covered the period from 01-10-2008 to 01-08-2012 and was updated (additional patients as well as updated data) till 01-09-2017 (1). For the current study all patients with available formalin-fixed paraffin-embedded (FFPE) tissue samples were selected. This study was approved by the ethics committee of Maastricht UMC+ (METC 2017-0318) and the need for informed consent was waived.

### 2.1 Data collection

The in-and outpatient medical records of all patients were retrieved. Eligible patients were patients with metastatic NSCLC, with data regarding molecular analysis and follow-up and sufficient FFPE tumor tissue available. The following data were collected: demographics, date of diagnosis of metastatic NSCLC, smoking status, histology, mutation status, site of biopsy l (e.g. pathology obtained from bone, lung, lymph node, adrenal lesion), baseline bone metastasis, development of bone metastases during treatment, treatment, skeletal related events (SREs) and time of death. SREs were defined as pathological fracture, spinal cord compression, necessity for radiation to bone (for pain or impending fracture) or surgery to bone (15).

### 2.2 Measurement of EGFR, RANKL, RANK and OPG gene expression

EGFR, RANKL, RANK and OPG expression was measured by reverse transcriptase quantitative real time Polymerase Chain Reaction (RT-qPCR) on ribonucleic acid (RNA) extracted from FFPE tissue. Data were presented as relative mRNA levels calculated by the equation 2^- delta cycling time (Ct)^. Delta CT is CT of target gene minus CT of housekeeping gene. Data were expressed on a logarithmic scale. See **Supplementary Material** for a more detailed explanation of the measurement of gene expression.

### 2.3 Statistics

Statistical analysis was conducted with SPSS (v20; SPSS Inc., Chicago, IL) and SAS 9.4. Descriptive statistics of demographic and clinical variables were obtained. Categorical variables were compared using chi-square tests and continuous variables were compared using the Mann-Whitney U Test or the Kruskal-Wallis test. Reverse Kaplan-Meier was used for calculating median follow-up time. Due to small sample sizes, bone metastases at baseline or development of bone metastases during disease were grouped together and classified as “bone metastases present”. EGFR gene expression was represented in quartiles, as there is no standard cut-off for high or low EGFR gene expression.

Competing risk analysis was used for the association between RANKL: OPG ratio and time to development of bone metastases for patients without bone metastases. The proportional hazards assumption was tested using time-dependent Cox regression analyses with interaction between RANKL: OPG ratio and time. Due to violation of this assumption the analysis was separated in two time intervals and the -2LogLikelihood was compared between models with different time-cut-off points to identify the best cut-off (i.e., the model with the lowest -2LogLikelihood).

The relation of EGFR, RANK, RANKL, OPG gene expression, RANKL: OPG ratio and bone metastases was the primary endpoint of this study. Secondary endpoints were 1) Association between sample origin (primary site, non-bone metastasis, metastasis in general except bone, bone) and expression of EGFR, RANK, RANKL and OPG and RANKL: OPG ratio, 2) Expression of EGFR, RANK, RANKL and OPG and RANKL: OPG ratio in different molecular subgroups (*EGFR+*, *KRAS+*, *EGFR*/*KRAS* wildtype) in relation to bone metastases.

## 3 Results

### 3.1 Patient characteristics

**Figure 1:**
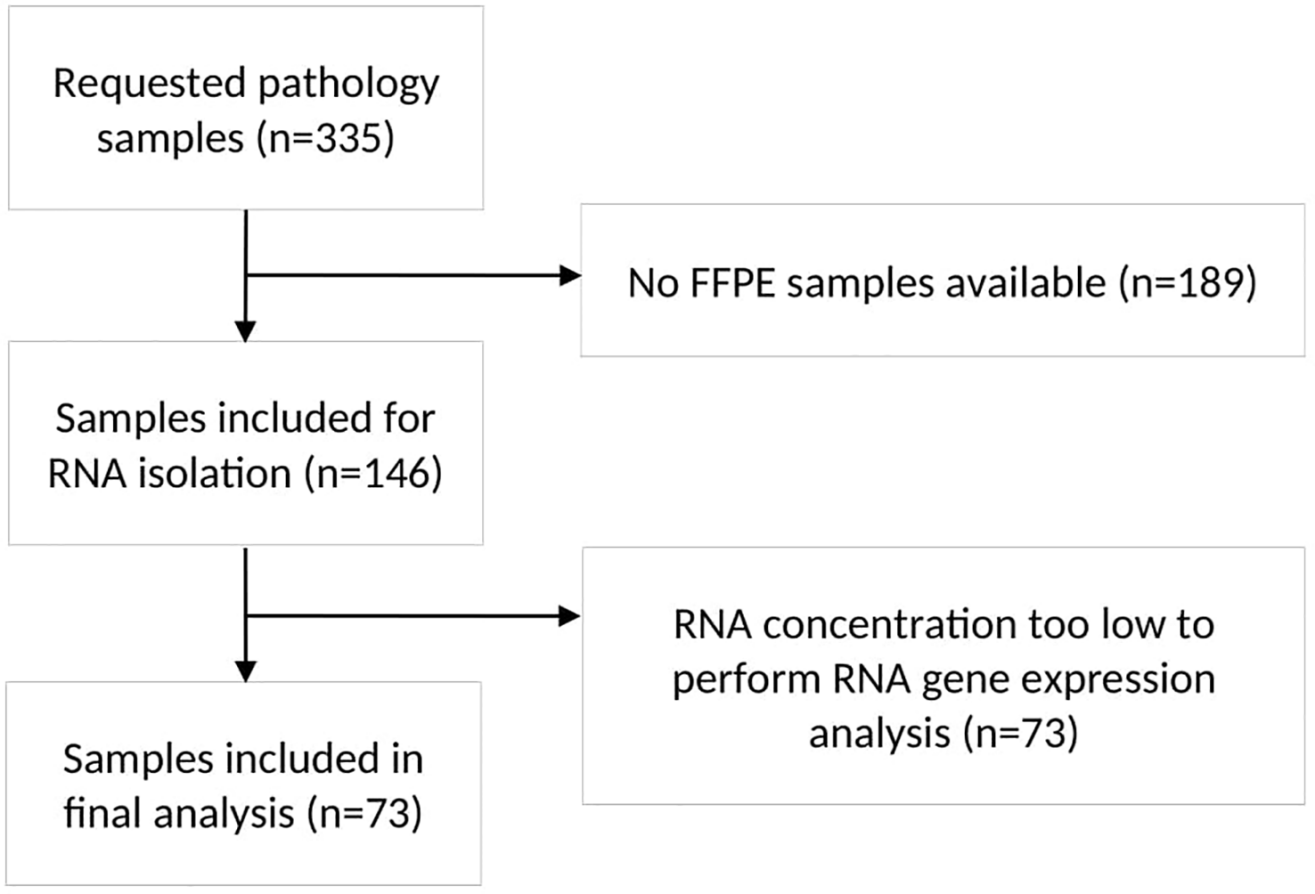
Flowchart of pathology sample selection. The flowchart showed the process of sample selection and reasons for exclusion of samples. n, number; FFPE, formalin-fixed paraffin-embedded; RNA, Ribonucleic acid.

From 169 patients (50%) of the total group of 335 patients, FFPE tumor samples were available. Ultimately, sufficient RNA could be extracted from 73 samples (Flowchart in **Figure 1**). In 52 out of 73 patients (81%), the pathology samples were obtained at diagnosis of metastatic disease. The other 21 patients were primarily diagnosed with early-stage NSCLC and had a median time to detection of metastatic disease of 550 days (range 87-2196 days). Patient characteristics are shown in **Table 1**. Median follow-up from diagnosis of metastatic NSCLC was 58.5 months (95% confidence interval (CI): 34.8-82.2 months).

**Table 1:** Patient characteristics.

Characteristics	Total n=73
Female n (%)	46 (63)
Never smoker n (%)	8 (11)
Mean age at diagnosis metastatic NSCLC, years (range)	62.8 (32-84)
Molecular subgroup n (%)	
*EGFR+* *KRAS*+ *EGFR*/*KRAS* wildtype	23 (32) 36 (49) 14 (19)
Tumor origin n (%)	
Lung (primary tumor) Bone Other metastasis	29 (40) 9 (12) 35 (48)
Metastatic disease at diagnosis n (%)	47 (64)
Bone metastases at diagnosis stage IV n (%)	27 (37)
Bone metastases at diagnosis or during course of disease n (%)	46 (63)
SRE n (%)^*^	26 (57)
Type of SRE n (%)^#^	
Radiotherapy Pathologic fracture Surgery Spinal cord compression	25 (96) 4 (15) 6 (23) 2 (8)
BTA use in all patients n (%)^$^ Denosumab Bisphosphonate	9 (12) 1 (1) 8 (11)

n, number; EGFR+, Epidermal Growth Factor Receptor mutation; KRAS+, Kirsten rat sarcoma mutation; SRE, skeletal related event; BTA, bone targeted agent.

^*^Percentages were calculated by group of patients with bone metastases.

^#^Percentages were calculated by subgroup of all pts with SREs (n=26). Some patients experienced more than one SRE.

^$^Denosumab was used in one patient without bone metastases, all patients who used bisphosphonates had bone metastases.

### 3.2 EGFR, RANKL, RANK, OPG gene expression

**Figure 2:**
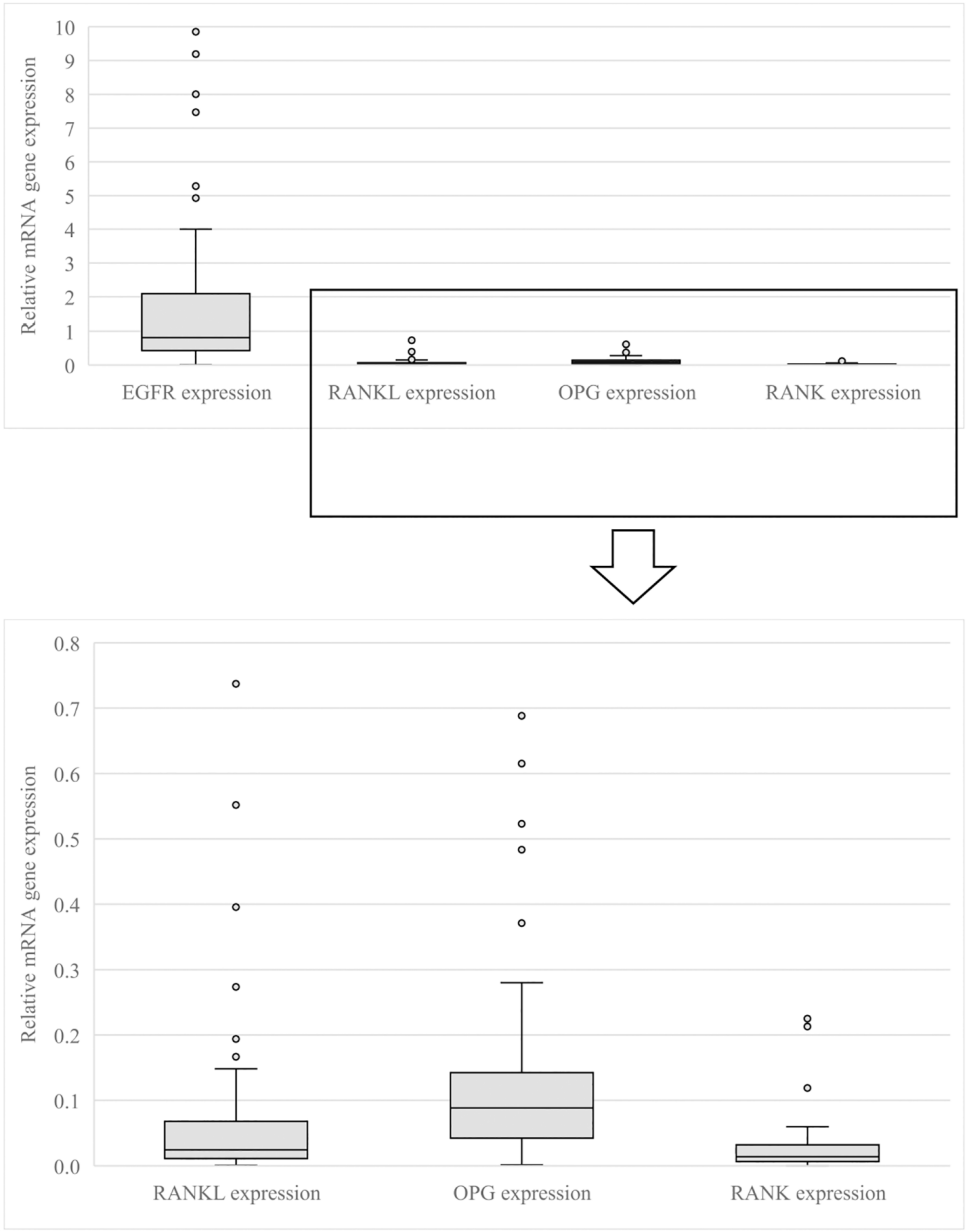
EGFR, RANKL, OPG, RANK gene expression in all patients. This figure shows the relative EGFR, RANKL, OPG and RANK gene expression measured on pathology samples of all patients. EGFR, Epidermal Growth Factor Receptor; RANKL, Receptor Activator of Nuclear Factor κB ligand; OPG, osteoprotegerin; RANK, Receptor Activator of Nuclear Factor κB.

EGFR, RANKL, RANK and OPG gene expressions were non-normally distributed (data not shown). The median EGFR expression was 0.84 (interquartile range (IQR) 1.67), the median RANKL expression was 0.02 (IQR 0.05), the median OPG expression was 0.09 (IQR 0.10) and the median RANK expression was 0.02 (IQR 0.03) (**Figure 2**).

### 3.3 Association between EGFR gene expression and RANKL, RANK and OPG gene expression or RANKL: OPG ratio and presence of bone metastases

**Figure 3:**
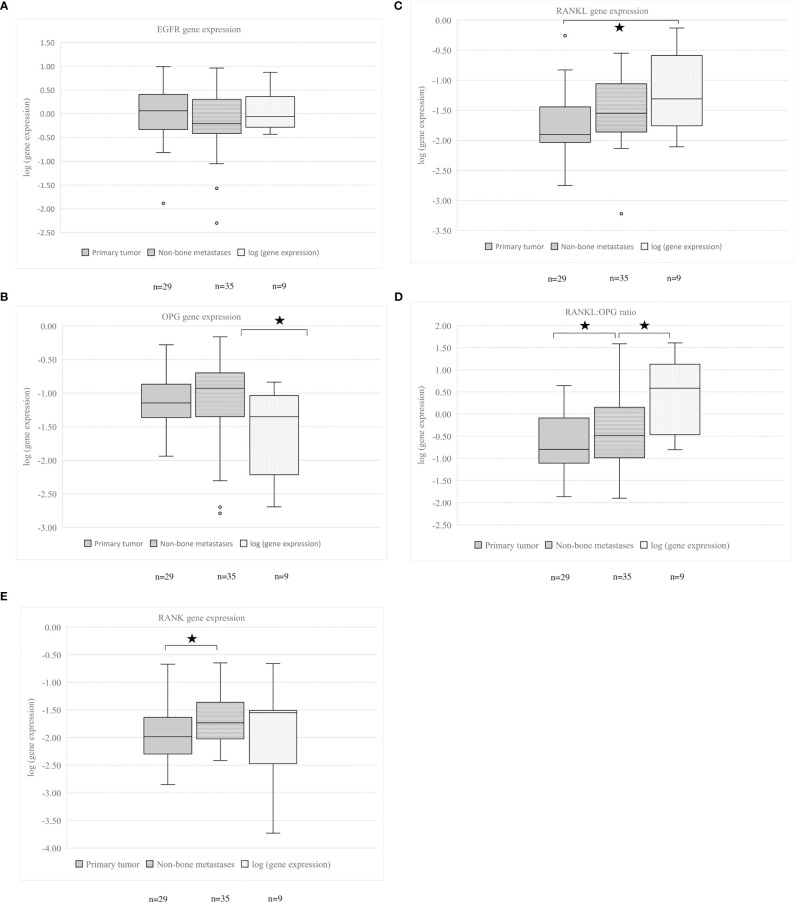
**(A–E)** EGFR, OPG, RANKL gene expression, RANKL: OPG ratio and RANK gene expression in relation to presence of bone metastases. Patients were subdivided in groups by EGFR expression. The first quartile is the lowest and the fourth quartile is the highest EGFR gene expression. **(A)** EGFR gene expression, **(B)** OPG gene expression, **(C)** RANKL gene expression, **(D)** RANKL: OPG ratio, **(E)** RANK gene expression. An asterisk denotes a significant difference between groups. EGFR, Epidermal Growth Factor Receptor; OPG, osteoprotegerin; RANKL, Receptor Activator of Nuclear Factor κB ligand; RANK, Receptor Activator of Nuclear Factor κB.

EGFR expression was similar for patients with and without bone metastases (p=0.479). The percentage of patients with and without bone metastases was comparable between all EGFR quartiles (p=0.174, **Figure 3A**). Patients with bone metastases had an increased tumor RANKL expression and increased RANKL: OPG ratio, compared to those without bone metastases (p=0.002 and p=0.026 respectively).

Subdividing patients based on EGFR quartiles showed that RANKL gene expression was numerically higher in all EGFR quartiles for patients with bone metastases and statistically higher in the second and third EGFR quartile (**Figure 3C**). In the different EGFR quartiles, no significant differences for OPG, RANK gene expressions and RANKL: OPG ratio and presence of bone metastases were observed (**Figures 3B, D, E**).

### 3.4 EGFR, RANKL, RANK and OPG gene expression or RANKL: OPG ratio in primary tumors and metastases

**Figure 4:**
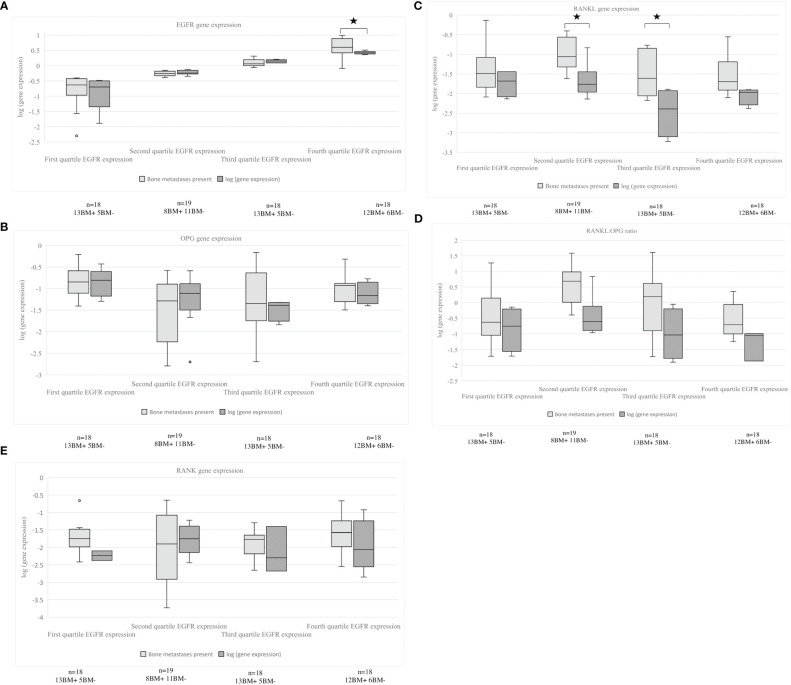
**(A–E)** EGFR, RANKL, OPG gene expression, RANKL: OPG ratio and RANK gene expression in relation to site of tumor biopsy. **(A)** EGFR expression, **(B)** OPG expression, **(C)** RANKL expression, **(D)** RANKL: OPG ratio, **(E)** RANK expression, all expressions are shown in primary tumor, non-bone metastases and bone metastases. An asterisk denotes a significant difference between groups. EGFR, Epidermal Growth Factor Receptor; OPG, osteoprotegerin; RANKL, Receptor Activator of Nuclear Factor κB ligand; RANK, Receptor Activator of Nuclear Factor κB.

The obtained tumor samples were subdivided based on site of origin: primary tumor (n=29), non-bone metastases (e.g., lymph node, liver, adrenal grand, parietal pleura, brain, other; n=35) and bone metastases (n=9). No difference was found for the percentage of tumor cells in the pathology sample and RANKL gene expression (data not shown). For the whole population of patients with and without bone metastases, significantly higher RANKL gene expression was observed in bone samples than in samples derived from the primary tumor (p=0.025). Pathology samples of both non-bone as well as bone metastases had a significant higher RANKL: OPG ratio in comparison to samples of the primary tumor (p=0.004 and p=0.028). The OPG gene expression was significantly lower in samples of bone metastases compared to non-bone metastases (p=0.043). RANK gene expression was significantly higher in samples of non-bone metastases in comparison to the primary tumor (p=0.047). **Figure 4** shows the different gene expression of the pathology samples. In the group of patients with bone metastases, no significant differences were observed between the various sample origins, only a trend to significance for OPG gene expression and RANKL: OPG ratio (p=0.072, p=0.079).

### 3.5 Gene expression of EGFR, RANKL, RANK and OPG or RANKL: OPG ratio in different NSCLC molecular subgroups in relation to bone metastases

Independent of the presence of bone metastases, patients with an *EGFR* mutation had a significantly higher EGFR expression, compared to patients with a *KRAS+* or *EGFR/KRAS* wildtype NSCLC (p<0.001) (**Supplementary Material, Figure 1A**).

Patients with *KRAS*+ NSCLC and bone metastases had a significantly higher RANKL expression and higher RANKL: OPG ratio (p=0.002) compared to patients with *KRAS+* NSCLC without bone metastases (p=0.017). This was not found for the other molecular subgroups. The OPG expression was significantly higher for patients with bone metastases in the subgroup of patients with *EGFR+* and *EGFR/KRAS* wildtype NSCLC (p=0.021 and p=0.028) (**Supplementary Material, Figures 1B-E**). No significant difference was observed between the different expression levels and presence of SREs in patients with bone metastases (data not shown).

### 3.6 Association between RANKL: OPG ratio and time to development of bone metastases

The RANKL: OPG ratio in relation to bone metastases development violated the proportional hazards assumption, therefore an early and late effect was determined. The hazard ratio (HR) of the RANKL: OPG ratio in the first 450 days after diagnosis of metastatic NSCLC was 1.65 (95% CI: 0.66-4.12) and decreased to 0.17 (95% CI: 0.03-0.95) thereafter.

## 4 Discussion

Previously, we showed that bone metastases were more frequent in patients with *EGFR+* metastatic NSCLC than in patients with *KRAS+* or *EGFR/KRAS* wildtype NSCLC, and that post bone metastases survival was significantly longer in patients with *EGFR+* NSCLC (1, 14). Based on preclinical data, showing that EGFR expression inhibits OPG expression and increases RANKL expression (5, 6), we hypothesized that the earlier observed increased EGFR gene expression in *EGFR+* NSCLC (8, 9) may lead to an altered shift of RANKL expression or RANKL: OPG ratio and thereby promote bone metastases in *EGFR+* NSCLC. In the current study, we indeed found that *EGFR+* NSCLC had a significantly higher EGFR gene expression as compared to *KRAS+* or *EGFR/KRAS* wildtype NSCLC. We could not demonstrate any association between EGFR gene expression level and the presence of bone metastases. However, patients with bone metastases had a significantly higher RANKL expression and RANKL: OPG ratio compared to those without bone metastases; possibly because the bone microenvironment in those with bone metastases released cytokines or growth factors which induced RANKL expression also in the tumor. This increased RANKL and RANKL: OPG ratio is in line with observations in an *in vitro* study in three human NSCLC cell lines and in 127 NSCLC tumor samples (52 primary tumors and 75 bone metastasis samples) in which the expression of RANKL, RANK and OPG was estimated by RT-PCR in cell lines and by immunohistochemistry (IHC) on tumor tissue (16). In addition, both *in vitro* and *in vivo* an increased RANKL expression and elevated RANKL: OPG ratio was associated with an enhanced potential of NSCLC to metastasize to the bone (16). Our data confirmed that patients with NSCLC with a higher RANKL: OPG ratio more often developed bone metastases, primarily in the first 450 days after diagnosis of metastatic NSCLC. Various studies have been performed to investigate biomarkers related to bone metastases or skeletal related event development. Examples are bone specific alkaline phosphatase in serum, urine N-terminal telopeptide in urine and C-X-C- Motif Chemokine Receptor 4 on the tumor (17). However, most of these are not used in daily practice as there is no recognized standard because of inconsistent study results. We showed an increased RANKL expression especially in patients with *KRAS+* NSCLC and bone metastases. As far as we know, no data about RANKL expression in this subgroup exists. Human lung adenocarcinoma data sets only showed that RANKL expression was significantly higher in *KRAS+* lung adenocarcinoma compared to *KRAS* wildtype lung adenocarcinoma (18).

As previously reported in breast or renal cell carcinoma, RANKL triggers the migration and metastasis of RANK expressing cancer cells (19, 20). A retrospective analysis in patients with non-metastatic breast cancer (n=509) showed a positive association between higher RANKL serum levels (measured by enzyme linked immune sorbent assay [ELISA]) and presence of disseminated tumor cells in the bone marrow and also with the development of bone metastases (21). Moreover, patients within the highest quartile of RANKL had a 4.6 increased risk for developing bone metastases compared to those within the lowest quartile (21). This is in line with our observation that patients with bone metastases had higher RANKL expression, especially in *KRAS+*NSCLC. It is not known whether the effect of RANKL inhibition (e.g., denosumab) on bone metastases related outcomes in patients with high versus low RANKL expression is different. In the Splendour trial no survival benefit was found when denosumab was added to first-line chemotherapy in patients with metastatic NSCLC (2). However, these patients were unselected for the presence of bone metastases and bone related outcomes were not reported. It would be of interest to explore the outcomes in patients with bone metastases and evaluate whether there is a relation between bone metastases related outcomes and RANKL expression (tumor or serum) as well as RANKL/OPG ratio (2).

In the current study, we could not find an explanation for our previously observed higher incidence of bone metastases in patients with *EGFR+* NSCLC (14). Although EGFR gene expression was higher, no association with a higher RANKL gene expression or RANKL: OPG ratio in tumor samples was observed. It could be that the tumor tissue is not the correct place to measure these values. Nowadays, more and more studies point on the role of extracellular vesicles (EVs) in bone metastases development in multiple types of cancer (22–24). An *in vitro* study showed that CRL-2868 NSCLC cells containing an *EGFR* 19 deletion, secrete exosomes containing EGFR ligand and Amphiregulin. These EVs were able to induce *in vitro* osteoclast differentiation of murine RAW264.7 cells by activation of EGFR phosphorylation and induction of matrix metalloproteinase-9 and tartrate-resistant acid phosphatase expression. These results were confirmed *ex vivo* by the finding that patient derived EVs were able to modulate osteoclastogenesis in human osteoclast precursors (23). Therefore, future studies should also focus on EVs in patients with (*EGFR+*) NSCLC to unravel the biological mechanism of bone metastases formation.

This study has its limitations. First, due to unavailability of tumor samples or impossibility to perform the gene expression analysis, the sample size was not large enough to have sufficient power for subgroup analysis. A second limitation is the different origin of the pathological samples, which could create bias in expression analysis as, by nature, RANKL expression in bone is higher than in lung tissue (25). However, as we had only nine bone samples in our analysis, we think this did not significantly affected our results. Third, not all patients underwent a 2-deoxy-2-[fluorine-18] fluoro-D-glucose positron emission tomography-computer tomography scan (FDG-PET-CT scan) or bone scintigraphy, therefore it could be that presence of bone metastases is underestimated as not all asymptomatic bone metastases will have been diagnosed by regular computed tomography of the chest and upper abdomen. Finally, patients with bone metastases and development of bone metastases during disease were grouped together and in doing so, one can ask whether the biological behavior of the tumor is the same in both groups. However, when analyzing both groups separately, the results remained similar (data not shown).

In conclusion, our study showed no association between EGFR gene expression and presence of bone metastases in patients with NSCLC; however, patients with bone metastases had a higher RANKL gene expression and RANKL: OPG ratio. An elevated RANKL: OPG ratio was associated with a higher incidence of bone metastases development, especially in the first year after diagnosis of metastatic NSCLC.

## Data availability statement

The raw data supporting the conclusions of this article will be made available by the authors, without undue reservation.

## Ethics statement

The studies involving human participants were reviewed and approved by Medisch-ethische toetsingscommissie Maastricht. Written informed consent for participation was not required for this study in accordance with the national legislation and the institutional requirements.

## Author contributions

AB: conceptualization, methodology, validation, formal analysis, investigation, writing-original draft, writing - review & editing, visualization, funding acquisition. LH: conceptualization, methodology, writing - review & editing, supervision, funding acquisition. IR-VB: conceptualization, methodology, validation, formal analysis, investigation, writing - review & editing. AD: formal analysis, writing - review & editing. GR: conceptualization, methodology, resources, writing - review & editing. BLJH: investigation, methodology, writing - review & editing. ZD: investigation, writing - review & editing. BH: investigation, methodology, writing - review & editing. DL: methodology, writing - review & editing. LM: methodology, writing - review & editing. RL: resources, writing - review & editing. MW: recourses, writing - review & editing. MD: conceptualization, methodology, validation, writing - review & editing, supervision. E-JS: conceptualization, writing - review & editing. A-MD: conceptualization, writing - review & editing, supervision, funding acquisition. All authors contributed to the article and approved the submitted version.
