# Supplementary material for: Association of RANKL and EGFR gene expression with bone metastases in patients with metastatic non-small cell lung cancer
Source: Front Oncol. 2023 May 5;13:1145001. doi: 10.3389/fonc.2023.1145001 (PMC10196450; doi:10.3389/fonc.2023.1145001)
Supplement: Supplementary file 1 [file DataSheet_1.pdf]

Supplementary Material

Association of RANKL and EGFR gene expression with bone metastases in patients with metastatic Non-Small Cell Lung Cancer

Anita J.W.M. Brouns, Lizza E.L. Hendriks, Iris J. Robbesom-van den Berge, J.H.M. Driessen, Guido M.J.M. Roemen, Britt L.J. van Herpen, Zoë Dekkers, Bas Heitzer, Daphne Leunissen, Laura Moonen, Ragnar Lunde, Marcel Westenend, Marjolein van Driel, Ernst-Jan M. Speel, Anne-Marie C. Dingemans*.

*** Correspondence:** Anne-Marie C. Dingemans: a.dingemans@erasmusmc.nl

1 Supplementary Materials and methods

1.1 EGFR gene expression

RNA was extracted from FFPE tissue using the automated Maxwell® RSC RNA FFPE Kit (AS1440; Promega USA) and subsequently quantified using the Quantifluor RNA System (E3310; Promega USA) on a Quantus Fluorometer (E6150; Promega USA).
Multiplex one step RT-qPCR were designed to determine the EGFR RNA expression levels. Importin 8 (IPO8) and Polymerase II polypeptide A (POLR2a) were used as reference genes to control the variability of clinical samples [25]. The expression of EGFR was determined one reaction: IPO8, POLR2a and EGFR. One step RT-qPCR analysis was performed (2 µl (1-40 ng) RNA input/reaction) on a Bio Rad Cfx96 instrument using the TaqPath™ 1-Step Multiplex Master Mix (No ROX) (A28522; Thermo Fisher Scientific USA) according to the manufacturer instructions, 400 nM of each primer and 100-150nM of each probe (Table 1, supplementary data 1). Normal lung tissue served as a positive control for EGFR expression.

1.2 OPG, RANKL and RANK gene expression

RNA was reverse transcribed into cDNA, using the SuperScript™ VILO™ cDNA Synthesis Kit according to the protocol of the manufacturer (Invitrogen, Carlsbad, California, United States). RT-qPCR was conducted using the Quantstudio Flex 7 system (Thermo Fisher Scientific, Waltham, Massachussetts, United States). Reactions were performed in 10 µl volumes using a SYBR green mastermix (GoTaq® qPCR Master Mix by Promega, Madison, Wisconsin, United States) in a 384 wellsplate (MicroAmp® Optical 384-Well Reaction Plate with Barcode, Applied biosystems®, Waltham, Massachussetts, United States). Reaction mixes contained 1 µl cDNA, 5 µl SYBR green mastermix, primers and RNAse free water. Primer sets were designed using the Primer-BLAST tool by the National Center for Biotechnology Information of the U.S. National Library of Medicine and manufactured at Integrated DNA Technologies (IDT), Coralville, Iowa, United States (Table 1). Cycling conditions were 50°C for two minutes, 95°C for 10 minutes, followed by 40 cycles of 95°C for 15 seconds and 60°C for 1 minute. Data are presented as relative mRNA levels calculated by the equation $2^{-\text{ delta cycling time (CT)}}$. Delta CT is CT of target gene minus CT of housekeeping gene (36B4).

2 Supplementary Figures and Tables

Table 1: Primer and probe-sequences.

RANKL	• Forward-primer: 5' GATGGTGGATGGCTCATGGT 3'<br>• Reverse-primer: 5' GGAACCAGATGGGATGTCGG 3'<br>• Probe: 5' [FAM] TCTGGCCAAGAGGAGCAAGC [BHQ1] 3'
EGFR	• Forward-primer: 5'GCAGCGATACAGCTCAGACC 3'<br>• Reverse-primer: 5'CTTTTGGGAACGGACTGGTTT 3'<br>• Probe: 5'[FAM] CGCCTTGACTGAGGACAGCA [BHQ1] 3'
RANK	• Forward-primer: 5' GTACCACTGGAGCCAGGACT 3'<br>• Reverse-primer: 5' CTTGTTGAGCTGCAACGGGT 3'
OPG	• Forward-primer: 5' CCATGTTCGTGGCCCTCC 3'<br>• Reverse-primer: 5' TAGGATCCATCTGCGCTCTG 3'
Housekeeping genes	
IPO8	• Forward-primer: 5' CGTTCCTCCTGAGACTCTGC 3'<br>• Reverse-primer: 5' TGCAGTGCCCACTTCTTACA 3'<br>• Probe: 5' [HEX] TGATAGACCAGAACTGGTATGGTGGA [BHQ1] 3'
POLR2a	• Forward-primer: 5'GCATTGACTTGCGTTTCCA 3'<br>• Reverse-primer: 5' TGCCGTTCCACCTTATAGCC 3'<br>• Probe: 5'[Cyanine 5] CCCAGTGACCTTCACCTGCA [BHQ3] 3'
36B4	• Forward-primer: 5' GTCCTCGTGGAAGGCCC 3'<br>• Reverse-primer: 5' AGGAGAGACAGGGAGCTCAG 3'

Abbreviations: RANKL: Receptor activator of NF- κB ligand; EGFR: Epidermal Growth Factor Receptor; RANK: Receptor activator of NF- κB, OPG: osteoprotegerin, IPO8: Importin 8; POLR2a: Polymerase II polypeptide A.

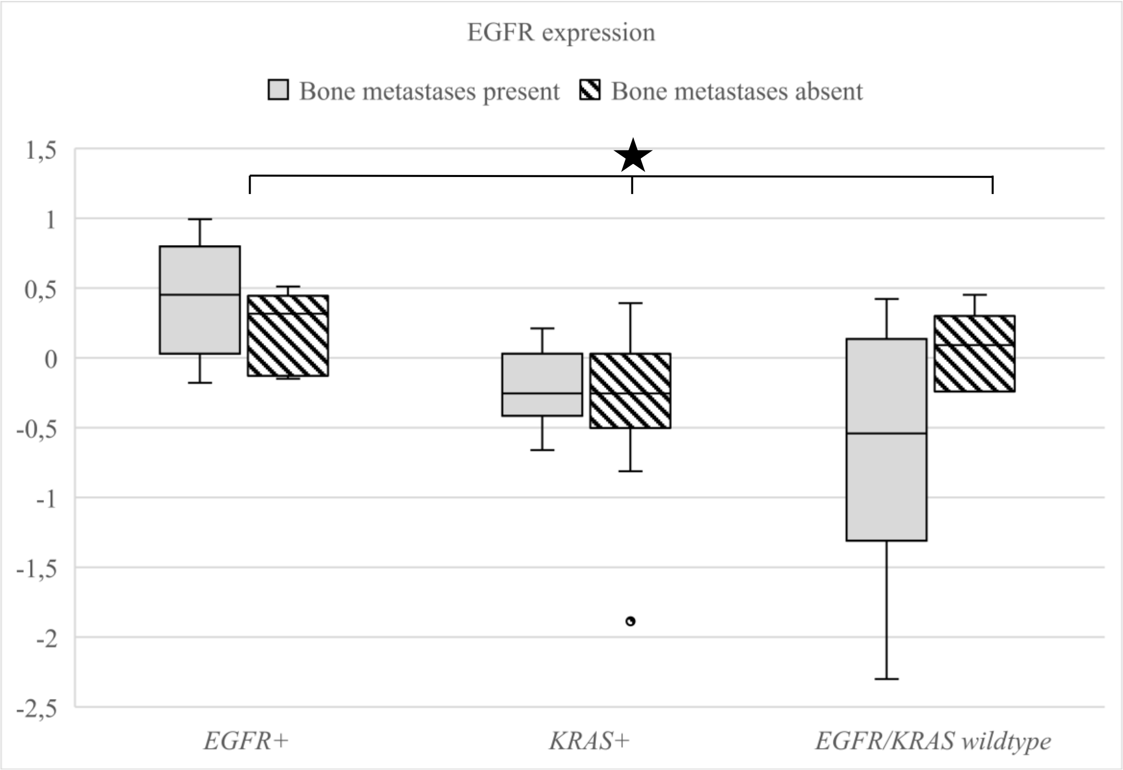

A)

n=23
17BM+ 6BM-

n=36
20BM+ 16BM-

n=14
9BM+ 5BM-

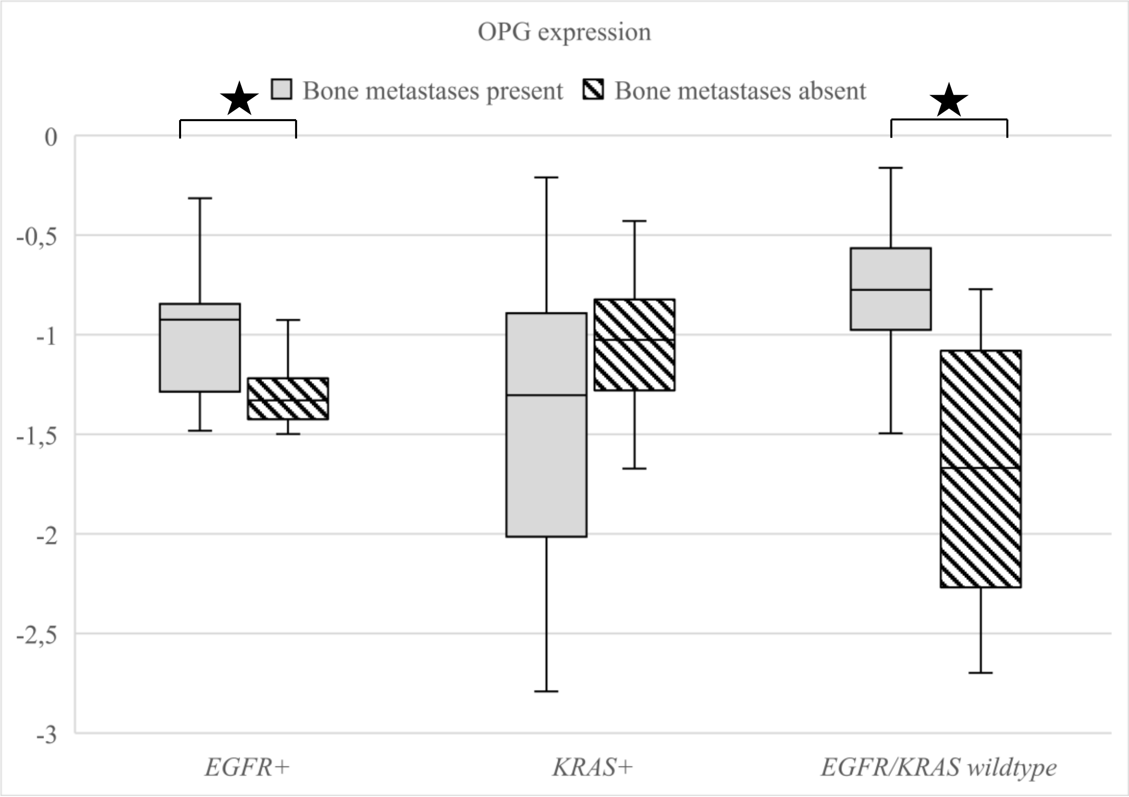

B)

n=23
17BM+ 6BM-

n=36
20BM+ 16BM-

n=14
9BM+ 5BM-

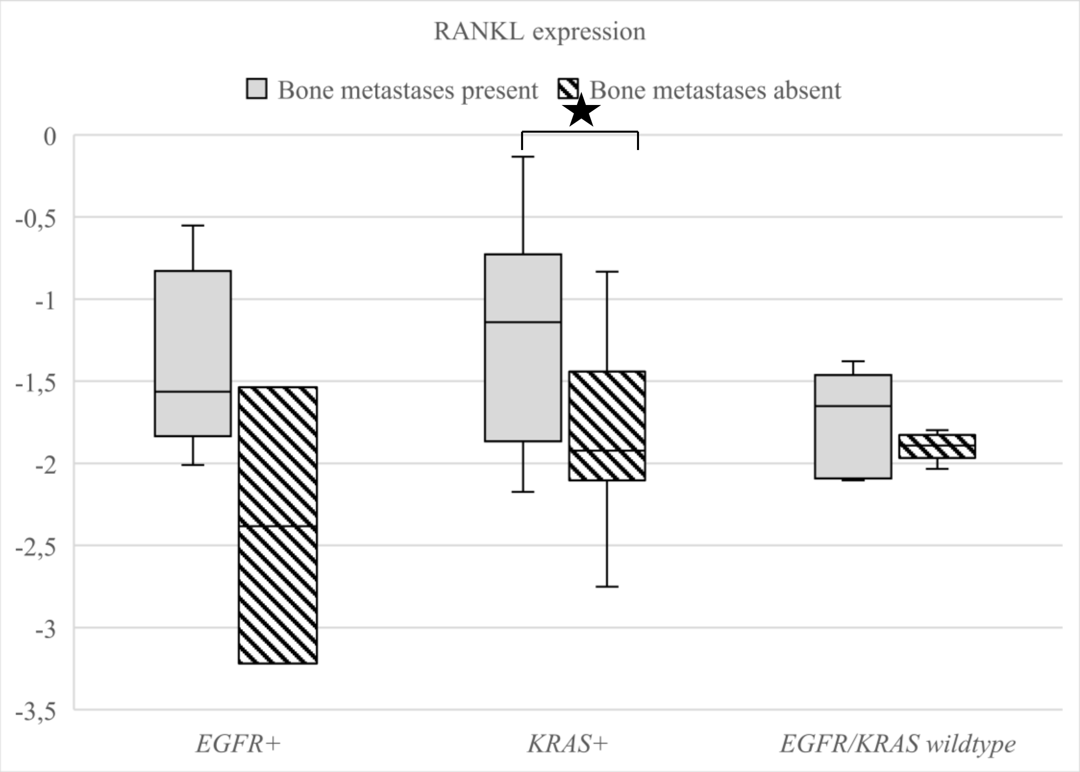

C)

n=23 17BM+ 6BM-

n=36 20BM+ 16BM-

n=14 9BM+ 5BM-

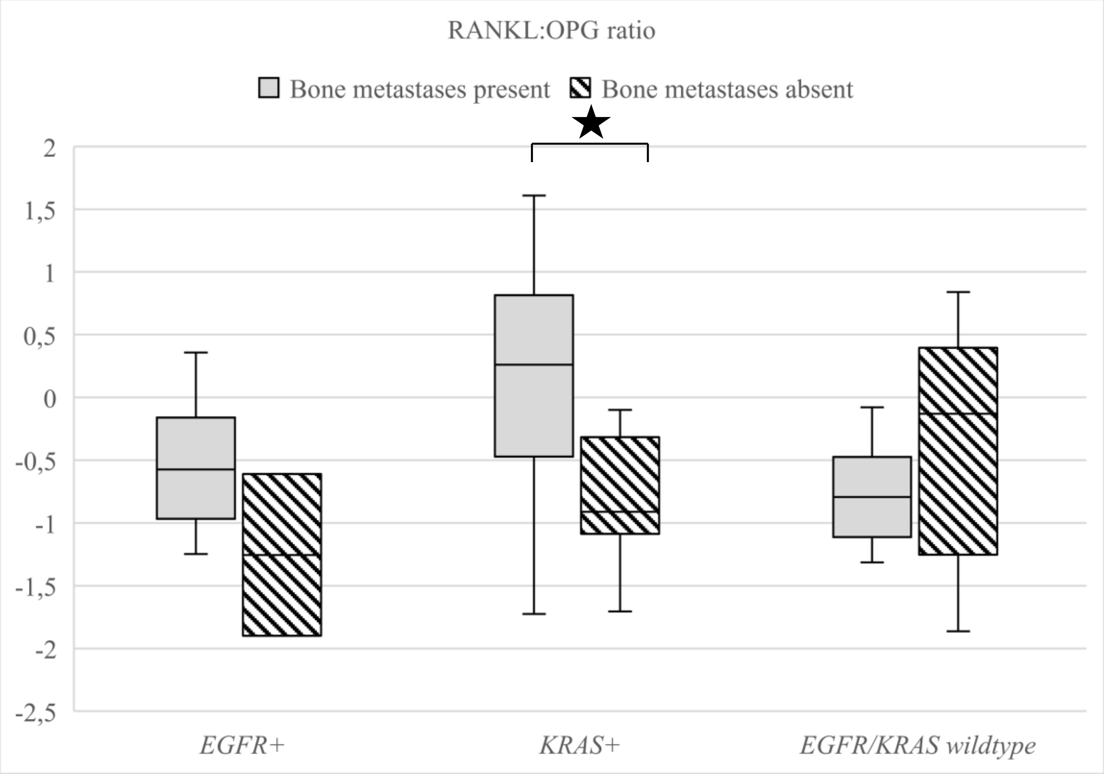

***D*)**

n=23 17BM+ 6BM-

n=36 20BM+ 16BM-

n=14 9BM+ 5BM-

E)

n=23
17BM+ 6BM-

n=36
20BM+ 16BM-

n=14
9BM+ 5BM-

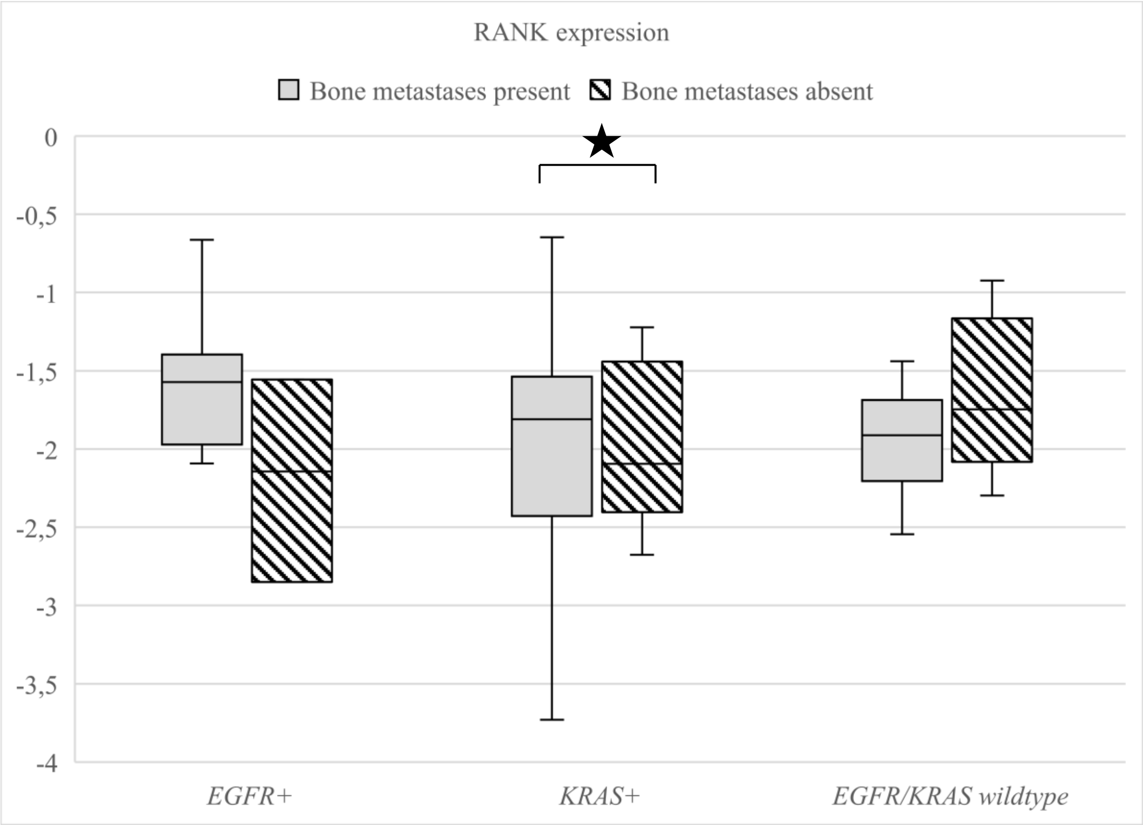

Figure 1 A-E: Relation between EGFR, RANKL, RANK and OPG expression or RANKL:OPG ratio and molecular subgroup.
A) EGFR expression, B) OPG expression, C) RANKL expression, D) RANKL:OPG ratio, E) RANK expression. An asterisk denotes a significant difference between groups.
Abbreviations: EGFR: Epidermal Growth Factor Receptor; OPG: osteoprotegerin; RANKL: Receptor Activator of Nuclear Factor κB ligand; RANK: Receptor Activator of Nuclear Factor κB.
